# Three different strategies to overcome dilation failures of the bougie cap for upper gastrointestinal refractory strictures

**DOI:** 10.1055/a-2081-9329

**Published:** 2023-05-15

**Authors:** Marie Laugié, Jérôme Rivory, Alexandru Lupu, Florian Rostain, Pierre Lafeuille, Clara Yzet, Mathieu Pioche

**Affiliations:** Gastroenterology and Endoscopy Unit, Edouard Herriot Hospital, Hospices Civils de Lyon, Lyon, France

Benign strictures of the upper gastrointestinal tract usually require endoscopic dilation either with balloons or bougies ( Fig. 1 ) as the first-line treatment. Both techniques are equivalent in terms of efficacy and safety 1 , but the use of bougies has a lesser environmental impact 2 . Compared to the reusable bougies, the BougieCap (Ovesco, Tübingen, Germany) allows for visual control during dilation 3 and has proven its effectiveness and safety in patients with eosinophilic esophagitis 4 with a low weight of disposable waste.

**Fig. 1:**
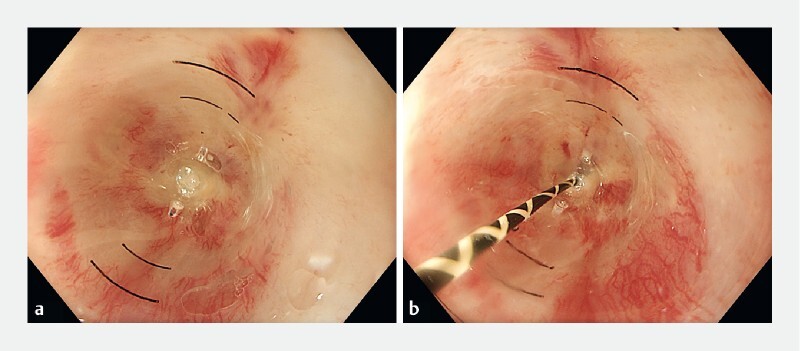
Bougie cap dilation. **a** Without guidewire. **b** With guidewire.

We report first a case of a second dilation attempt in a 60-year-old man with a pharyngeal stenosis following submucosal dissection for squamous cell carcinoma. Dilation with a 12-mm BougieCap and guidewire pushed through the cap was impossible. We dilated with a 12-mm balloon and thereafter passed a 13.5-mm BougieCap ( Video 1 ).

**Video 1** Three failures of bougie cap dilation: salvage strategies to achieve dilation.


We next report a case of two esophageal refractory strictures following a caustic ingestion. We succeeded in dilating the first stenosis, which was short at 10 cm from the dental arches, with a 14-mm BougieCap, but the second stricture was longer at 30 cm and could not be crossed with the 14-mm BougieCap. We dilated with a 13-mm Savary-Gilliard bougie and then we used a 14-mm BougieCap again to pass the stricture.

The last case was a refractory post-endoscopic submucosal dissection (ESD) stricture treated by dilation 3 months ago with a 15-mm dilation balloon. We first tried to pass the 15-mm bougie cap but were unsuccessful. We failed again with the 13/14-mm bougie cap device, but the 12-mm cap passed through the stricture and allowed successful consecutive dilation with the 13/14 and the 15/16 mm.

For certain strictures, the use of the BougieCap seems compromised and alternative strategies should be available to dilate the stricture when the bougie cap fails.

Endoscopy_UCTN_Code_TTT_1AO_2AH

## Notes

Endoscopy E-Videos

https://eref.thieme.de/e-videos
*E-Videos* is an open access online section of the journal *Endoscopy* , reporting on interesting cases and new techniques in gastroenterological endoscopy. All papers include a high-quality video and are published with a Creative Commons CC-BY license. Endoscopy E-Videos qualify for HINARI discounts and waivers and eligibility is automatically checked during the submission process. We grant 100% waivers to articles whose corresponding authors are based in Group A countries and 50% waivers to those who are based in Group B countries as classified by Research4Life (see: https://www.research4life.org/access/eligibility/ ). This section has its own submission website at https://mc.manuscriptcentral.com/e-videos
